# The Behavioural Economics of Music: Systematic review and future directions

**DOI:** 10.1177/17470218221113761

**Published:** 2022-08-16

**Authors:** Manuel Anglada-Tort, Nikhil Masters, Jochen Steffens, Adrian North, Daniel Müllensiefen

**Affiliations:** 1 Audio Communication Group, Technische Universität Berlin, Berlin, Germany; 2 Computational Auditory Perception Group, Max Planck Institute for Empirical Aesthetics, Frankfurt am Main, Germany; 3 Department of Economics, School of Social Sciences, The University of Manchester, Manchester, UK; 4 Hochschule Düsseldorf, University of Applied Sciences, Düsseldorf, Germany; 5 School of Population Health, Curtin University, Perth, WA, Australia; 6 Department of Psychology, Goldsmiths, University of London, London, UK; 7 Hanover University of Music, Drama and Media, Hanover, Germany

**Keywords:** Behavioural economics, music, decision-making, systematic review, interdisciplinary research

## Abstract

Music-related decision-making encompasses a wide range of behaviours including those associated with listening choices, composition and performance, and decisions involving music education and therapy. Although research programmes in psychology and economics have contributed to an improved understanding of music-related behaviour, historically, these disciplines have been unconnected. Recently, however, researchers have begun to bridge this gap by employing tools from behavioural economics. This article contributes to the literature by providing a discussion about the benefits of using behavioural economics in music-decision research. We achieve this in two ways. First, through a systematic review, we identify the current state of the literature within four key areas of behavioural economics—heuristics and biases, social decision-making, behavioural time preferences, and dual-process theory. Second, taking findings of the literature as a starting point, we demonstrate how behavioural economics can inform future research. Based on this, we propose *the Behavioural Economics of Music* (BEM), an integrated research programme that aims to break new ground by stimulating interdisciplinary research in the intersection between music, psychology, and economics.

## Introduction

Music is a human universal with ancient origins, present in every known culture worldwide (Conard et al., 2009; Mehr et al., 2019; Savage et al., 2015). Activities involving music, such as music listening and performance, are central to the human experience and for many people represent an important part of everyday life (DeNora, 2000). This article focuses on *music-related decision-making*—judgements and decisions associated with music, for which music is of primary interest.^1^ Specifically, we discuss the benefits of applying behavioural economics to music-decision research. To this end, we conduct a systematic literature review with the primary goal of identifying studies that have utilised behavioural economics to examine music-related decision-making. Our second goal is to explore how behavioural economics can be used for future research. From these two objectives, we propose *the Behavioural Economics of Music* (BEM), an interdisciplinary research programme that promotes the study of music-decision research using the tools of behavioural economics.

Our motivation flows from research within psychology and cognitive science (often referred to as music psychology and music cognition), aimed at understanding the psychological processes involved in music experience and behaviour (see Deutsch, 2013; Hallam et al., 2016; Honing, 2017; North & Hargreaves, 2008; Tan et al., 2017, for general overviews). This work provides a rich literature in key areas of music-related decision-making and has been crucial in advancing our knowledge of the processes that govern music-related behaviours (Anglada-Tort & Sanfilippo, 2019). For example, pleasurable listening experiences have been linked to the reward-processing areas of the brain (see Belfi & Loui, 2020, for a review) and shown to fulfil several psychological needs, such as self-awareness, emotional regulation, and social connectedness (see Schäfer et al., 2013, for a review). Playing music and dancing to rhythmic sounds with others have also been found to create and strengthen social bonds, through the release of neurohormones such as endorphins (see Savage et al., 2021, for a review). Moreover, cognitive responses to music interact with other human functions including movement, speech, attention, and memory, insights that have become crucial for furthering advances in music education and therapy (see MacDonald et al., 2013, for a review).

Independent from psychology, there is an established but smaller literature examining music-related decision-making through the lens of economics (see Byun, 2016; Cameron, 2016; Connolly & Krueger, 2006; Tschmuck, 2017, for general overviews). A key feature of the work undertaken by economists is the analysis of music decisions using tools from neoclassical economics, primarily based upon optimisation of economic agents within consumer theory, producer theory, and game theory. Examples include music consumption and piracy (see Montoro-Pons et al., 2021; Oberholzer-Gee & Strumpf, 2010; Varian, 2005, for reviews), competitive dynamics in the music industry (Burke, 1996; Ko & Lau, 2016; Sweeting, 2013), superstardom and commercial success (Hamlen, 1991, 1994; Hendricks & Sorensen, 2009; Ordanini, 2006; Strobl & Tucker, 2000), and the economics of live music events (Courty & Pagliero, 2014; Hiller, 2016; Krueger, 2005).

While there is clear evidence that research in psychology and economics has both contributed significantly to an improved understanding of music-related behaviour, only relatively recently have researchers begun to utilise tools from behavioural economics to study music-related decision-making. Behavioural economics increases the explanatory power of neoclassical economics by relaxing the assumptions of *homo economicus*—that is, individuals are perfectly rational, self-interested, pursuing goals of utility maximisation. Instead, from the viewpoint that individuals are limited in their rationality, behavioural economics has developed a body of theory incorporating insights from an array of disciplines, including psychology, sociology, anthropology, biology, and neuroscience (see Angner & Loewenstein, 2012; Cartwright, 2018; Dhami, 2016; Thaler, 2016, for overviews). The behavioural economics toolkit has been successfully applied to other complex domains including health (Blumenthal-Barby & Krieger, 2015; Rice, 2013), education (Jabbar, 2011), environmental policy (Brekke & Johansson-Stenman, 2008; Frederiks et al., 2015), and politics (Sunstein & Thaler, 2003; Sunstein, 2014). Informed by the recent literature, our contribution is to demonstrate the added value of applying this interdisciplinary approach to music decision-making research.

To provide an example, consider how musicians make decisions while improvising or how a listener selects which song to play next when streaming music. In both cases, individuals may use mental shortcuts or *heuristics* to come to a decision quickly rather than spend hours considering all the myriad alternatives. As another example, consumers may choose to make voluntary payments for music, even when they have the opportunity to acquire it for free. This could be explained by theories of *social preferences*, whereby individuals care about the preferences of others as well as their own, incorporating concerns for reciprocity, altruism, and fairness. These brief examples illustrate the synergistic benefits of applying behavioural economics to music-decision research from the perspective of both psychologists and economists. In short, relaxing the rationality assumptions and incorporating interdisciplinary insights, allow for a more empirically-supported approach. At the same time, utilising behavioural economic models provides an internally consistent body of theory to work within. This is the essence of our proposed BEM research programme.

Using a robust search strategy, we identified studies related to behavioural economics and music-decision-making within four distinct research areas—heuristics and biases, social decision-making, behavioural time preferences, and dual-process theory. We organise our discussion of the literature around these areas, which also provides the structure for our proposals for future research. The remainder of this article is organised as follows. We next outline the methods for the systematic literature review. We then give an overview of the results followed by a more in-depth discussion of the retrieved literature. Finally, we discuss future work alongside introducing the BEM research programme.

## Methods

This section outlines the methods employed for the systematic literature review. We first present our procedure for selecting the behavioural economics keywords used in the systematic search. We then give details of each stage of the systematic review.

### Behavioural economics keywords

**Figure 1.**
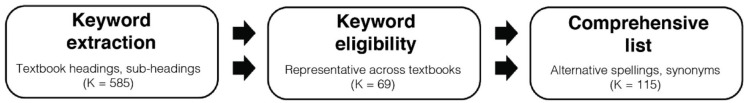
Behavioural economics keywords selection. K = number of keywords at each step in the selection procedure.

An important requirement for our objectives is to select a list of keywords for the systematic search that is representative of behavioural economics. Summarised in Figure 1, this procedure consisted of three steps: (1) extraction of keywords appearing in chapter headings and subheadings of prominent textbooks in behavioural economics and decision-making published within the last 10 years, resulting in 585 keywords across all sources (Angner, 2016; Ball & Thompson, 2017; Cartwright, 2018; Dhami, 2016; Hastie & Dawes, 2010; Holyoak & Morrison, 2012; Ogaski & Tanaka, 2017; Wilkinson & Klaes, 2017); (2) assessing keyword eligibility by selecting only those that were representative across multiple textbooks (i.e., repeated in at least two of the textbooks), leaving 69 keywords; and (3) creation of a comprehensive list by including alternative spellings (e.g., behaviour vs. behaviour) and synonyms (e.g., mental accounting vs. psychological accounting), adding 46 extra keywords. The final list comprised a total of 115 keywords (see the online Supplementary Material for the complete list).^2^

### Systematic literature review

**Figure 2.**
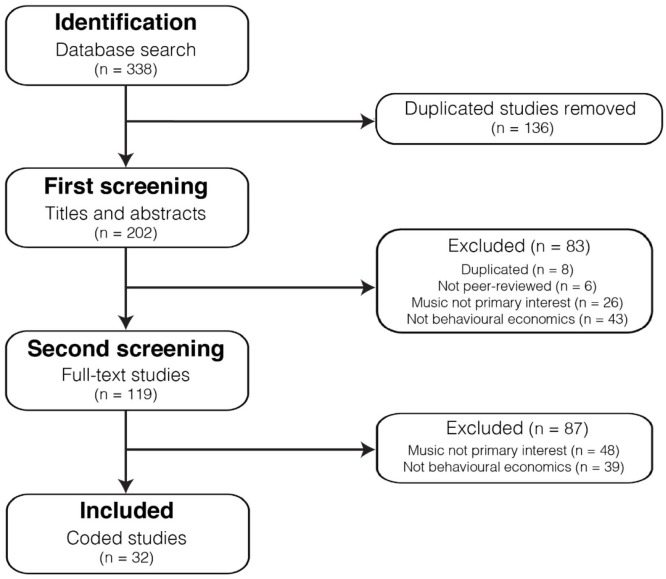
PRISMA flow diagram.

Following an established protocol, we applied the methodology outlined by the Preferred Reporting Items for Systematic Reviews and Meta-analyses (PRISMA; Moher et al., 2009). The systematic review consisted of four stages: (1) identification of studies through a database search, (2) first systematic screening based on titles and abstracts only, (3) second systematic screening based on full text, and (4) coding of the final set of included studies. Figure 2 summarises the outcome of each stage using a PRISMA flow diagram.

In the identification stage, a database search was conducted using the list of 115 behavioural economics keywords connected with the keyword “music” (including possible variations such as musical, musicality, musicians, musicianship). The search was undertaken in June 2018 using Scopus, Web of Science, PsycINFO, Academic Search Complete, Business Source Complete, and Google Scholar. The search strategy for all databases was identical, searching for the syntax in the title, abstract, or authors’ keywords. The search was limited to peer-reviewed journals published in English. In this first stage, 338 studies were identified, which after duplicate studies were removed, resulted in 202 studies ready for screening.

In all systematic screenings, we used the following inclusion criteria to determine whether a study was included:

1. The study is written in English and is published in a peer-reviewed journal.
2. The study examines judgements and decision-making related to music, where music is of primary interest.
3. The study applies behavioural economics to music-related behaviour.

In the first systematic screening, two reviewers independently screened each study based on the title and abstract only (*n* = 202). The percentage agreement between reviewers was 80%, resulting in 41 cases resolved by a third reviewer. From this first screening, 83 studies were removed according to the inclusion criteria, resulting in 119 studies proceeding to the second screening. In the second screening, two reviewers independently assessed each study based on full text (*n* = 119). The percentage agreement between reviewers here was 70%, resulting in 36 cases resolved by the third reviewer. From this second screening, 87 studies were removed. The final number of studies included for discussion was 32.

Each study was independently coded by two reviewers along the following attributes: broad research area within behavioural economics, area studied within music, academic discipline (determined by journal published), and methods used (experimental, field data, survey, theoretical). A third reviewer resolved any disagreements in the coding.

## Overview of results

From the systematic literature review, we found 32 studies that applied behavioural economics to examine music-related decision-making. Based on the results of the coding, we categorised these studies into four main areas of behavioural economics (henceforth known as BEM areas): heuristics and biases (*n* = 15), social decision-making (*n* = 9), behavioural time preferences (*n* = 4), and dual-process theory (*n* = 4).

Table 1 presents summary information for each study organised by BEM area. Within music, the most common area studied was music consumption and piracy (*n* = 13), followed by music preferences (*n* = 9), music performance (*n* = 5), music perception and memory (*n* = 4), and music and health (*n* = 1). The studies came from a range of disciplines, with the majority from psychology (*n* = 15), economics (*n* = 10), neuroscience (*n* = 5), business (*n* = 1), and health (*n* = 1). The majority of studies were empirical (*n* = 27) with the most common method of data collection being experimental (*n* = 19), the rest being survey (*n* = 2), field data (*n* = 2), and mixed methods (*n* = 4). A small number of studies were theoretical (*n* = 5). Publication dates indicate that this literature is relatively recent (24 out of the 32 studies were published in the last 10 years). While we acknowledge that there may be studies that have used similar approaches, but have not been included in the review as they have not explicitly used behavioural economics terminology, we are confident that the set of studies identified here provides a clear snapshot of the current literature on the role of behavioural economics in music-decision research.

**Table 1.** Summary of the literature from the systematic review (*n* = 32).

Authors	Music area	Discipline	Methods	Key findings
Heuristics and biases				
*Judgement heuristics*				
Anglada-Tort et al. (2019)^a^	Music preferences	Psychology	Experimental	Participants gave lower ratings of aesthetic value to music, after song titles had been manipulated to evoke feelings of negative affect (*affect heuristic*).
Lonsdale and North (2012)	Music preferences	Psychology	Experimental	Participants based their evaluations of music tastes of other individuals on how similar these individuals were to stereotypical music fans (*representativeness heuristic).*
Li and Cheng (2014)	Music consumption and piracy	Business	Survey	*Status quo bias* identified as a factor that decreases consumer switching intentions from free to paid music streaming services.
Rozin et al. (2004)	Music perception and memory	Psychology	Experimental	Participants judged the emotional intensity of past music experiences upon how they felt at the most intense point and at the end, rather than the average of every moment during the piece (*peak-end rule*).
Schäfer et al. (2014)	Music perception and memory	Psychology	Experimental	Participants judged the emotional intensity of past music experiences using both the average of all experienced moments in the piece, as well as the peaks and the end (*peak-end rule*).
Vuvan et al. (2014)	Music perception and memory	Psychology	Experimental	Participants were more likely to falsely remember that a test tone was contained within a melody, when the tone was more musically related to the melody, and hence more easily brought to the mind (*availability heuristic*).
Watson et al. (2017)	Music consumption and piracy	Psychology	Survey	Perceived benefits of illegal music-file sharing were negatively related to the perceived risks, rather than being independent of each other (*affect heuristic*).
Processing fluency				
Anglada-Tort and Müllensiefen (2017)^b^	Music preferences	Psychology	Experimental	Repeated exposure to familiar pop music led participants to give increased preference ratings for that music, while repetition of less familiar classical music did not affect ratings.
Anglada-Tort et al. (2019)^a^	Music preferences	Psychology	Experimental	Through manipulation of artist names and song titles, identical music presented with easy-to-pronounce names were preferred compared with music presented with difficult-to-pronounce names.
Huron (2013)	Music performance	Psychology	Theoretical	Proposal of performance strategies that apply processing fluency so that musicians can maximise the overall hedonic effect of the performance.
Nunes et al. (2015)	Music consumption and piracy	Psychology	Experimental/field-data	Songs with more repetitive lyrics were perceived as more familiar and found to have an increased likelihood of being commercially successful.
Seror and Neil (2015)	Music perception and memory	Psychology	Experimental	Pitch discrimination was found to be faster and more accurate in consonant harmonic intervals than dissonant harmonic intervals.
Witvliet and Vrana (2007)	Music preferences	Psychology	Experimental	Repeated exposure to emotionally positive music led to increased liking for that music, whereas repetition of emotionally negative music led to increased disliking for that music.
*Framing effects*				
Anglada-Tort and Müllensiefen (2017)^b^	Music preferences	Psychology	Experimental	Participants evaluated identical recordings more positively when the music was framed as being performed by a professional musician rather than performed by a less skilled musician (prestige effects).
Aydogan et al. (2018)	Music preferences	Neuroscience	Experimental	Neuroimaging evidence indicated that higher activation in the vmPFC was able to explain the prestige effect. Increased activation in the dlPFC was able to explain suppression of this bias through cognitive control.
de Bruijn et al. (2016)^c^	Music and health	Health education	Experimental	Messages with consequences framed as losses were an effective strategy to reduce listening to music at high volume among adolescents.
North and Hargreaves (2005)	Music preferences	Psychology	Experimental	Identical pop songs either framed as “suicide-inducing” or “life-affirming” affected perceptions of the harmful nature of such music.
Social decision-making				
Social preferences				
Harbi et al. (2014)	Music consumption and piracy	Economics	Theoretical	Theoretical model examined the profitability of PWYW vs. fixed price. Under the assumption that consumers care about the welfare of the artist, PWYW can be profitable by promoting voluntary payment and higher prices for live performance.
Hashim et al. (2014)	Music consumption and piracy	Economics	Experimental	In a public good experiment framed in the context of music consumption, piracy among adolescents was reduced the most when they had received advice from sources that had the strongest social ties with them, and who could be punished for the actions of the participants.
Regner and Barria (2009)	Music consumption and piracy	Economics	Field data/theoretical	Customers buying music online under a PWYW agreement gave payments that exceeded the minimum payment. The proposed behavioural game-theoretical model indicated that reciprocity is able to explain these generous payments.
Regner (2015)	Music consumption and piracy	Economics	Field data/survey	Follow-up of Regner and Barria (2009) combining customer transaction data with a survey regarding buying motivations. The results indicated that reciprocity was a driver for generous payments.
Sonnabend (2016)	Music consumption and piracy	Economics	Theoretical	Theoretical model applied fairness concerns by fans about pricing for live music events. Such concerns can lead the artist to keep prices down, even if there are surges in demand.
Waskow et al. (2016)	Music consumption and piracy	Neuroscience	Experimental	Neuroimaging data indicated differences in neural activity for participants in a PWYW condition vs. fixed price.
Peer effects				
Berlin et al. (2015)	Music consumption and piracy	Economics	Experimental	Teenage participants were influenced by music ratings evaluated by peers, increasing the dominance of superstars in the music market.
Berns et al. (2010)	Music consumption and piracy	Neuroscience	Experimental	Neuroimaging evidence found that the desire for conformity was a driver for young people to change likability ratings of songs after receiving popularity information.
Berns and Moore (2012)	Music consumption and piracy	Neuroscience	Experimental/field data	Follow-up of Berns et al. (2010) found that neuroimaging data at the individual level can predict future music popularity at the market level.
Behavioural time preferences				
Charlton and Fantino (2008)	Music consumption and piracy	Economics	Experimental	Time preferences for music followed a hyperbolic function indicating that participants placed high value on immediate consumption.
de Bruijn et al. (2016)^c^	Music and health	Health education	Experimental	Messages containing short-term consequences were an effective strategy to reduce listening to music at high volume among adolescents, indicating that such individuals were susceptible to present bias.
Gans (2015)	Music consumption and piracy	Economics	Theoretical	Theoretical model explains why music artists continue to enter the industry amidst lost revenue from piracy. At the start of their careers, time-inconsistent artists under-weigh their preferences to “sell out” and earn money, and therefore are less concerned about the loss of any future revenue from piracy.
Kahneman and Snell (1992)	Music preferences	Economics	Experimental	Participants were found to be poor at predicting their future hedonic experiences of listening to music.
Kahnx et al. (1997)	Music preferences	Business	Experimental	When making repeated choices for songs to play, participants did not always choose song to maximise enjoyment, but instead opted for less-preferred music to seek variety.
Dual-process theory				
Bangert, Fabian, et al. (2014)	Music performance	Psychology	Field data	65% of musical decisions made by a professional cellist while performing a familiar piece were categorised as deliberate (System 2), compared with 35% intuitive (System 1).
Bangert, Schubert, and Fabian (2014)	Music performance	Psychology	Theoretical	Model describes how expertise can affect the interaction between intuitive and deliberate decision-making of music performers.
Bangert et al. (2015)	Music performance	Psychology	Field data	82% of musical decisions made by professional violinists while performing an unknown piece were categorised as intuitive, compared with 18% deliberate.
Rosen et al. (2016)	Music performance	Neuroscience	Experimental	Expertise moderated the effect of increased deliberative processing on the quality of jazz improvisations. Neurostimulation on the dlPFC increased the performance quality for less experienced musicians, but hindered the performance quality for expert musicians.

vmPFC: ventromedial prefrontal cortex; dlPFC: dorsolateral prefrontal cortex; PWYW: pay-what-you-want; BEM: Behavioural Economics of Music.

a ^b, c^These studies are duplicated in the table to indicate their categorisation into multiple BEM areas. This categorisation is also reflected in the discussion in the next section.

## Discussion of the literature

In this section, we provide an in-depth discussion of the retrieved literature with specific focus on the application of behavioural economics within each study. We organise this discussion around the BEM areas outlined in the previous section.

### Heuristics and biases

When evaluating music, listeners employ *heuristics* to inform their decisions. Heuristics are mental shortcuts used by individuals to simplify complex decisions into easier to calculate operations, allowing people to make these decisions quickly and efficiently (Kahneman et al., 1982; Tversky & Kahneman, 1974). The use of heuristics represents a departure from neoclassical economics, which assumes that individuals are fully rational utility maximisers who have limitless cognitive abilities and follow the laws of probability and statistics. Instead, research has shown that people use information selectively, which can be useful when fast decision-making is required, but can also lead to suboptimal decisions, known as cognitive biases (see Dhami, 2016; Hastie & Dawes, 2010, for reviews).

With nearly half of the studies identified applying heuristics and biases to music decision-making, this was found to be the most common area of research. As such, we further divide this section into the following subsections—judgement heuristics, processing fluency, and framing effects.

#### Judgement heuristics

Several studies concerning judgements about music have shown that people apply heuristics using information recalled from memory, such as the availability heuristic and the peak-end rule. The *availability heuristic* describes the tendency for individuals to judge the likelihood of an event by the ease with which similar events can be brought to mind (Tversky & Kahneman, 1974). Vuvan et al. (2014) examined whether the availability heuristic can explain tonal expectancies in music memory. Participants were presented with melodies followed by a test tone and subsequently asked to indicate whether the test tone was present in the melody. Test tones were manipulated to either be expected or unexpected to occur in the melody, based upon their relatedness to the melody in terms of tonality and scale. The authors found that participants falsely recalled that the test tone was in the melody more frequently when it was highly expected to be in the melody, consistent with the idea that such tones are more easily “available” to the mind.

Rozin et al. (2004) and Schäfer et al. (2014) investigated how listeners make affective judgements about past music experiences. Both studies found that in line with the *peak-end rule* (Fredrickson & Kahneman, 1993), participants judged the emotional intensity of the music upon how they felt at its most intense point (the peak) and at its end, rather than the total sum of every moment of the experience.

Music judgements have also been found to be influenced by the emotionality of information presented with a song. Anglada-Tort et al. (2019) examined whether evaluations about the aesthetic value of music differed when song titles had been manipulated to evoke positive, negative, or neutral feelings. Song titles influenced listeners’ preferences, with music presented with negative titles receiving the lowest ratings in aesthetic value. This finding is consistent with the *affect heuristic*—the tendency to rely on the feelings experienced in relation to a stimulus when making judgements and decisions (Slovic et al., 2002).

Watson et al. (2017) investigated whether music piracy is motivated by the affect heuristic. From an analytical view, the perceived benefits and risk of an activity are qualitatively distinct from each other and not correlated. However, the authors found that participants’ judgement of the benefits of illegal music-file sharing (e.g., financial benefits, ease of access) was negatively related to their perception of risk (e.g., lawsuits against individuals), a common characteristic of the affect heuristic (see Finucane et al., 2000), giving support that individuals rely on affective judgements when evaluating music piracy.

Lonsdale and North (2012) found evidence that music stereotypes (i.e., how people judge the likely music taste of others) can be explained through the *representativeness* heuristic—the tendency to judge the probability that a sample belongs to a population by looking at the degree to which that sample resembles the population (Tversky & Kahneman, 1974). Specifically, the authors found that when asked to evaluate the music taste of another individual, participants’ judgements were highly correlated with how similar they perceived that individual to a stereotypical group (e.g., an individual described to engage in antisocial behaviour was more likely to be attributed to liking hip-hop music), rather than the base-rate probability estimates for being a fan of that genre.

Finally, in the context of online music streaming services, Li and Cheng (2014) analysed survey data to examine why consumers may be reluctant to switch from a “free” model, whereby consumers do not pay for music but the content contains advertising, to a “fee” model, whereby consumers receive higher-quality content with no advertising but pay a monthly subscription. The authors attribute this behaviour to the *status quo bias*—the tendency to stay with the current option (Samuelson & Zeckhauser, 1988). For example, they found that consumers were concerned about the perceived sacrifices of leaving the current plan including the monetary cost, the time and effort to switch, and the risk that the new plan would not be enjoyable.

#### Processing fluency

*Processing fluency* refers to the subjective experience of ease when processing information. A key observation from the literature is that more fluent stimuli are often perceived as more familiar and aesthetically pleasing than less fluent stimuli (see Reber et al., 2004, for a review). In particular, factors thought to influence fluency, such as repeated exposure to a stimulus (Zajonc, 1968) and complexity of information contained within a stimulus (Checkosky & Whitlock, 1973), have been shown to affect both music perception and evaluation.

Witvliet and Vrana (2007) investigated how fluency arising from repeated listening influenced participant liking of music. Using music pieces that varied by emotional valence, the results indicated that repeated exposure to emotionally positive music led to increased liking for that music, whereas repetition of emotionally negative music led to increased disliking for that music. Anglada-Tort and Müllensiefen (2017) found that the effect of repetition interacted with pre-existing familiarity: participants’ liking for music increasing with repeated listening only for familiar pop music and not for a relatively unknown classical piece. The results from both studies suggest that while repeated exposure has an influence on music preferences, factors associated with the listener experience such as emotional connection and familiarity with the music seem important to uncover the more nuanced patterns in this relationship.

Seror and Neil (2015) found that properties of the music itself affect fluency and music perception. In a pitch discrimination task, participants indicated whether a single note could be detected within a harmonic interval of several notes played simultaneously. Faster and more accurate pitch discrimination was identified within consonant intervals, associated with feelings of pleasantness and agreeableness, and perceived to be more fluent (e.g., a perfect fifth), rather than dissonant intervals, associated with unpleasantness and harshness, perceived as less fluent (e.g., a tritone).

To examine the effects of fluency arising from music complexity, some studies have altered the linguistic properties of music stimuli (linguistic fluency). In the laboratory, Nunes et al. (2015) manipulated the amount of repetition in the lyrics of otherwise identical songs and found that this was related to increased perceived familiarity. The authors then examined the effect of repetitiveness on a song’s popularity in the marketplace using field data from the U.S. singles chart. The results indicated that more repetitive songs were more likely to be number one hits and faster climbers to the top of the chart. Anglada-Tort et al. (2019) found that when manipulating the linguistic fluency of artist names and song titles in a foreign language, identical music excerpts presented with easy-to-pronounce names were preferred to excerpts presented with difficult-to-pronounce names. These results even held for participants with high levels of music training, indicating that susceptibility to the biasing effects of processing fluency is not offset by increased knowledge of music.

Finally, Huron (2013) discussed ways in which fluency can be used by musicians to increase the overall hedonic effect of music on an audience. Although repeated exposure can lead to favourable evaluations, too much repetition may lead to a counter-effect of habituation, in which listeners may eventually become unresponsive to the music. To address this, Huron proposes compositional strategies that differ to varying degrees in the amount of repetition within a music score versus the inclusion of new material. Such an approach gives an insight into how heuristics such as processing fluency can be applied practically by musicians.

#### Framing effects

*Framing* represents the systematic change in an individual’s decision when faced with normatively equivalent choices that differ in terms of the information presented (Tversky & Kahneman, 1981; see Kühberger, 1998, for a meta-analysis). Sources of framing could include a semantic manipulation of the choice or differences in the contextual information associated with that choice. In the context of music performance evaluation, several studies have identified *prestige effects*. Anglada-Tort and Müllensiefen (2017) found that listeners evaluated identical recordings more positively in terms of liking and quality when the music was framed as being performed by a professional musician rather than performed by a less-skilled musician. In a neuroimaging study, Aydogan et al. (2018) found that higher activation in the ventromedial prefrontal cortex (vmPFC), a region in the brain shown to play a key role in subjective value, was able to explain prestige effects observed for participants assessing music played by a student versus a professional musician. Interestingly, for participants who preferred the student performance, increased activation was observed in the dorsolateral prefrontal cortex (dlPFC), a region related to cognitive control and deliberative effortful thinking. This finding suggests that these participants were able to suppress the framing bias by exerting cognitive control.

An important area in which framing has been applied is the music decisions of adolescents. North and Hargreaves (2005) found that merely labelling music as being harmful to young people (suicide-inducing) affected perceptions of the deleterious nature of such music, compared with when labelling the same music using a positive frame (life-affirming). de Bruijn et al. (2016) applied framing to an intervention study to induce behavioural change among adolescents regarding hearing loss prevention. Young people recruited from schools initially provided information on their music listening behaviour including intentions to listen to music at low volumes. Two weeks later, they were then asked the same questions after they had been exposed to persuasive messages about hearing loss, framed as a gain-frame (positive consequences of listening to music at a reduced volume) versus a loss-frame (negative consequences of not doing so). The authors found that the loss-frame was an effective strategy to increase intentions. Such findings are consistent with *loss aversion* (Tversky & Kahneman, 1991), whereby risks framed as losses lead to behaviours to avoid this loss more proportionately than when framed as gains.

#### Summary

A number of studies have found that individuals apply heuristics to music-related decision-making, particularly in the context of probabilistic assessments and aesthetic judgement. These include the use of the availability heuristic and peak-end rule in making judgements about past musical experiences, the affect heuristic using emotions as a guide to decision-making, and the representativeness heuristic when considering music stereotypes. We also found a specific area of the literature dedicated to processing fluency, whereby repetition (both repeated listening and within a song) and music complexity can induce fluency to alter music perception and preference. Finally, studies have indicated that differences in how information associated with music is framed can influence music evaluation and be an essential tool for behavioural change among adolescents.

### Social decision-making

This section discusses literature from the review that has applied theories of social decision-making from behavioural economics. We find two streams of work—social preferences and peer effects.

#### Social preferences

In 2007, the critically acclaimed band Radiohead surprised the music industry by offering their new album “In Rainbows” as a digital download using a pay-what-you-want (PWYW) agreement. Essentially, this meant that fans could pay as much as they liked for the album, including a zero option. Although at odds with neoclassical economic theory, which predicts that consumers would simply download the album for free, fans actually made voluntary payments for the album. A possible explanation for such generous payments under PWYW is that individuals exhibit *social preferences*, that is, they care about the preferences of others (see Fehr & Schmidt, 2006, for a review). In this subsection, we outline the findings of studies that have examined PWYW schemes as well as some broader issues surrounding social preferences and music consumption.

An important tool used in behavioural economics to model social preferences is *behavioural game theory*. Like classical game theory, behavioural game theory considers strategic decision-making among multiple players, but unlike its classical counterpart, standard assumptions regarding self-interest and rationality are relaxed, allowing for models that are more empirically supported (see Camerer, 2003; Camerer & Ho, 2015; Dhami, 2016). Regner and Barria (2009) applied behavioural game theory to investigate motivations behind generous payments under PWYW. Initially, using field data from an independent record label, the authors found that around 85% of customers chose to make payments that exceeded the minimum required payment, with the average payment above the recommended price set by the label. The authors conjectured that since the label offers an extensive try-before-you-buy service, reciprocity may be driving the generous payments. More formally, using a theoretical model the authors show how concerns for reciprocity can switch behaviour from a selfish outcome, in which customers simply offer the minimum, to a more generous outcome, as observed in the data.^3^ In a follow-up study, Regner (2015) combined the transaction data with survey data regarding motivations behind customer transaction providing evidence that reciprocity was a driver for generous voluntary payments.

Another explanation for why consumers make payments under PWYW is that they gain procedural utility from buying music, that is, they care not only about their satisfaction from consuming music but also the conditions in which the music is made, including the welfare of the artist. Harbi et al. (2014) included this possibility in a theoretical model to compare artist profitability under PWYW versus a fixed-price scenario. The model demonstrated that a PWYW pricing strategy can be more profitable for the artist as it promotes positive voluntary payments reducing piracy as well as increasing demand for live performance through increased music coverage.

Waskow et al. (2016) explored whether differences in payments for albums under PWYW versus a traditional fixed price could be explained at the neural level. Consistent with the previous literature, payments in the PWYW condition were significantly greater than zero. Neuroimaging data revealed significant differences between the two conditions, with willingness-to-pay being related to reward processing in the frontal brain regions, but only in the fixed-price condition. No such relationships were found in the PWYW condition, indicating that the neural processes for voluntary payments of music may be distinct from when consumers pay a fixed price.

Sonnabend (2016) incorporated fairness concern (Fehr & Schmidt, 1999) into a theoretical model looking at pricing decisions of music artists in the live music industry. In the model, fans are concerned about the fairness of prices of live gigs, such that if prices are above a reference price, they do not buy tickets.^4^ In particular, these concerns can be enough for the artist to keep prices low, even at times when there is higher demand, for example, on the weekends. Higher prices are tolerated however, when due to increased costs borne by the artist, since they are perceived as fair by fans.

Finally, Hashim et al. (2014) examined the effectiveness of different sources of advice to reduce music piracy among adolescents. Piracy behaviour was investigated through a public goods experiment framed in the context of music consumption.^5^ In this game, each participant decided whether to buy songs or download them for free from other participants in the group. In addition, participants were assigned to treatments that differed in terms of the source of advice they would receive during the experiment. Contrary to the game-theoretic prediction of maximum piracy, in which participants free-ride off the others in the group, piracy behaviour in the experiment was found to be below this level, providing evidence of social preferences. Notably, piracy was reduced the most when the advice came from sources that had the strongest social ties with the participants, such as from parents, and when the adviser had a stake in the game and could be punished for the actions of the participants in the group.

#### Peer effects

There is considerable evidence that consumption choices made by an associative reference group can influence an individual’s decision to purchase a product (Bearden & Etzel, 1982; Bursztyn et al., 2014; Childers & Rao, 1992; Escalas & Bettman, 2005). Possible reasons include learning from peers’ choices to gain additional information (Banerjee, 1992; Bikhchandani et al., 1992) or simply the desire to conform (Cialdini & Goldstein, 2004). Regarding music consumption, peer effects have been shown to play a significant role for adolescents, who represent not only a group for which music is important (North et al., 2000), but one for which peer effects are prevalent (Steinberg & Monahan, 2007).

Berlin et al. (2015) found that music ratings evaluated by peers influenced teenagers’ song choices. In particular, more listening time was devoted to bestsellers rather than new artists, thereby strengthening the so-called “superstar effect,” whereby relatively small numbers of artists dominate the music industry.

In a neuroimaging study, Berns et al. (2010) found that the desire for conformity was a driver for young people to change likability ratings of songs after receiving popularity information based on the number of times each song had been played on a website. Specifically, the tendency to change one’s evaluation was correlated with neural activity in the bilateral anterior insula and anterior cingulate cortex, regions associated with negative feeling states, suggesting that for these participants, the mismatch between their ratings and others’ ratings may have led to cognitive/emotional dissonance that had to be resolved. In a follow-up study, Berns and Moore (2012) found that the same neuroimaging data could predict future sales of these songs, with activity within the ventral striatum (associated with reward) being correlated with future commercial success. Therefore, it appears that, in addition to the influence of social information on music consumption at the individual level, brain responses of individuals can predict future commercial success at the population level.

#### Summary

Much of the identified literature on social decision-making has examined the role of social preferences in music consumption. In particular, reciprocity, concern for an artist’s welfare, and attitudes around fairness are shown to be significant factors in determining how much consumers are willing to pay for music. We also found literature on peer effects in music consumption with evidence that adolescent music choices are influenced by the choices of others, with an indication that this is driven by the desire to conform.

### Behavioural time preferences

A significant amount of research in behavioural economics has been devoted to decisions that have a time dimension (see Dhami, 2016, for a review). A central aspect of this work is that individuals exhibit *present-biased* time preferences, that is, a strong preference for immediate gratification (O’Donoghue & Rabin, 1999). In some cases, this desire can be so strong that it can lead an individual to alter a previously made decision at a later point in time. Here, the standard exponential discounted utility model often assumed in neoclassical economics is insufficient to capture these patterns of time inconsistency, and instead a hyperbolic function is a more accurate representation.^6^ This section outlines the literature from the review that has applied behavioural time preferences, particularly in the domains of music consumption and hedonic value.

As previously discussed, De Bruijn et al. (2016) carried out a framing intervention study to examine adolescent behaviour surrounding hearing loss prevention. In addition to finding that persuasive messaging framed as losses increased student intentions to listen to music at low volumes, the study also investigated whether the temporal framing of consequences (short vs. long term) would affect behaviour. The authors found that only messages containing short-term consequences of loud music were effective in changing listening intentions. This finding suggests that the young people in the sample were susceptible to present bias by overweighting immediate negative consequences and underweighting the long-term consequences.

Several studies have used time preferences to examine the experiential value of music. Charlton and Fantino (2008) measured the temporal discount rate of various commodities (including music) by asking participants to choose between a given quantity of a commodity today versus $100 after some delay. The authors found that time preferences for music fitted a hyperbolic function well, indicating that participants placed high value on immediate consumption similar to other primary reinforcers such as food and drink.^7^ Kahneman and Snell (1992) investigated the ability to forecast future hedonic experiences from listening to music. Participants listened to the same piece of music for seven consecutive days after they had given predictions about how they would like the music after this time. The results indicated that the participants were poor at hedonic forecasting, overestimating the effect of repetition in reducing their future liking for the music. Kahnx et al. (1997) examined how individuals decide which songs to play over a given period of time, such as when creating a playlist. They found that when making repeated choices between a liked song and a less-preferred song, listeners did not always choose the song that maximised their enjoyment but instead opted for less-preferred music to seek variety.

Finally, Gans (2015) applied behavioural time preference modelling to address an ongoing question in the music industry: Why are artists still entering the music industry if revenue from selling music has decreased due to digital technology and piracy? In the model, he proposed that artists face a dynamic trade-off between fame (the intrinsic reward of being supported by fans) and fortune (the revenue generated from music sales). So, although new artists may initially choose fame to build up a fan base, they may choose to “sell out” in the future to focus on financial rewards. Crucially, by allowing for time-inconsistent preferences, the model shows that when starting out, artists under-weigh the idea that they will sell out in the future, and therefore are not deterred by the threat of lost future revenue due to piracy.

### Dual process theory

*Dual process theories* posit that there are two modes of processing—an emotional and a cognitive system. The emotional system (System 1) is fast, automatic, and unconscious, whereas the cognitive system (System 2) is slow, deliberative, and conscious (see Evans, 2008; Frankish & Evans, 2009, for reviews). Exploring the interaction between emotional and cognitive processes has been particularly insightful for research in music performance, in which studies have identified factors that determine whether performers rely on conscious versus unconscious decisions.

Bangert, Fabian, et al. (2014) conducted language analysis of retrospective accounts from an expert cellist performing a piece of familiar music, with the goal of understanding which music decisions were intuitive and which were deliberate. The results indicated that out of the 134 decisions made, 65% were categorised as deliberate. In a second study, Bangert et al. (2015) applied the same method using a small sample of professional violinists, but with an unfamiliar piece of music. This time, 82% of music decisions were categorised as intuitive. Taken together, these results suggest that familiarity of music is an important factor in determining whether performers apply System 1 or System 2 processing.

Another factor thought to determine how performers switch between intuitive versus deliberate decision-making is expertise. Bangert, Schubert, and Fabian (2014) present a theoretical representation, known as the “spiral” model. The model proposes that when a novice starts, they rely on intuition stemming from less developed knowledge (immature intuition), with the performance likely to contain mistakes. After practising, the performer increases their knowledge and moves towards a process of greater deliberation based upon more informed decisions. With even more practise, the performer returns to intuitive processing, but this now comes from highly developed knowledge, so that deliberate decisions have become automatic (mature intuition). As the performer encounters new music problems, each iteration between intuitive and deliberate decision-making contains fewer mistakes, with the performer having greater control. In a neurostimulation study, Rosen et al. (2016) test whether expertise can moderate the effect of increased deliberative processing on the quality of jazz improvisations. Transcranial direct current stimulation (tDCS) was applied to the dlPFC (a region related to deliberative thinking) of jazz pianists of varying expertise. The results indicated that the stimulation increased the performance quality for less experienced musicians, but hindered the performance quality for expert musicians. These findings are consistent with the spiral model suggesting that novices benefit from increased top-down control, whereas experts benefit from heightened intuitive processing.

## Future directions

Having demonstrated the value of behavioural economics to music-decision research using the current literature, we now turn to our second objective of exploring how behavioural economics can inform future research. Again, to guide our discussion, we use the BEM areas categorised from the systematic review. In the final part of this section, we introduce the BEM research programme and discuss how researchers can develop its potential.

### Heuristics in music performance

The area of heuristics and biases was found to be the most prevalent research topic within the review, focusing almost exclusively on issues related to music preferences and consumption. Surprisingly, however, we found no studies that applied heuristics to music composition and improvisation choices. Given the highly demanding nature of music improvisation, including both cognitive and bodily limitations (Ashley, 2016), it seems likely that musicians rely on fast and frugal heuristics to simplify complex decisions while improvising. A recent study by Beaty et al. (2021) provides some initial evidence for this, demonstrating that eminent jazz musicians tend to start their solo improvisations with music sequences that are melodically simpler before creating more complex sequences, the so-called “easy-first” bias. We hypothesise that musicians may be relying on other heuristics in their performances, some of which have not yet been studied in the music domain at all. One such candidate is the *anchoring heuristic* (Tversky & Kahneman, 1974), whereby musicians during a performance rely on initial musical choices (the anchor) to inform future decisions. At this point, we note the link between heuristics and the two-system view from dual process theory (Kahneman, 2011). If performers are using heuristics and applying System 1 processing to save on cognitive effort, then this may be observable at the neural level. In this regard, we encourage further use of the research methods employed in the field of improvisation neuroscience (see Beaty, 2015, for a review), to gain a deeper understanding of the neural processes that underpin heuristics in music composition and performance.

### Cognitive biases in music consumption

A potentially fruitful application of cognitive bias research is *choice overload*. The dramatic increase in recent years of music streaming services (e.g., Spotify, YouTube) provides listeners with a large assortment of songs instantly. However, listeners may not necessarily be benefitting from this vast amount of choice. In fact, much evidence indicates that providing individuals with variety can lead to negative outcomes including choice deferral, choice reversal, reverting to the default option, and overall lower satisfaction (see Chernev et al., 2015, for a review). Despite voluminous research on choice overload, there has been little application to music listening behaviour. One promising avenue of research is provided by Ferwerda et al. (2019), who found that music expertise moderates the relationship between how music is organised on streaming platforms (e.g., mood, genre, activity) and preferences for the size of choice set. For example, when presented with music organised by mood, participants with more music expertise preferred a system with fewer choices, but when presented with music organised by genre, these participants preferred a system with more choices. We see scope to build on this work to understand more fully how individual differences influence music taxonomy choices, minimising the adverse effects of choice overload and improving the user experience from music streaming services.

### Social decision-making and piracy

As discussed in the review, social preferences can lead individuals to make voluntary payments for music, while peer effects can influence an individual’s consumption choices. We see benefit in combining these areas of research to examine ways to reduce music piracy. For example, a robust finding in laboratory experiments is that many people are “conditional co-operators,” whose pro-social behaviour is sensitive to observations of others’ behaviour (see Chaudhuri, 2011, for a review). Exploring conditional cooperation in a music setting may therefore hold the key to increasing compliance associated with music payments. In addition, to further understand the dynamics of music consumption and emergence of social norms in a more naturalistic environment, we encourage the use of social network experiments (see Hawkins et al., 2019, for a review). Such methods could be used to model the complex cognitive processes involved in music consumption, such as learning, social coordination, and cultural transmission.

### Behavioural game theory and hit song science

We see a great amount of potential in applying behavioural game theory further to music-decision research. One area of advance is hit song science, a field aimed at predicting song success before market release. Traditionally, attempts at predicting song popularity have only considered the intrinsic properties of the music itself, but given that social factors have a substantial influence on market outcomes, this approach has been unsuccessful (see Pachet, 2012). One novel way to model social influence is to use *Level-k and cognitive hierarchy models* (Camerer et al., 2004; Stahl & Wilson, 1994, 1995). In such models, there is a hierarchy about what players believe about the actions of other players.^8^ For example, a well-known application for which Level-*k* modelling has made accurate predictions about behaviour is the beauty contest (Keynes, 1936; Nagel, 1995). Here, we consider a music adaptation, where individuals have to guess the song which corresponds to the average preference of the competition. While Level 0 individuals may simply choose their favourite song, Level 1 players will choose the song that they believe the majority of Level 0 players will choose, and Level 2 players will choose a song incorporating their beliefs about Level 1 players, and so on. Understanding how individuals form beliefs in music prediction markets and how these beliefs are affected by others could give greater insight into how songs become popular, potentially increasing prediction accuracy in hit song science.

### Behavioural time preferences in music education

The review indicated that present-biased preferences can lead an individual to reverse a previously made decision at a later point in time. Since time-inconsistent preferences are often detrimental to the long-term interest of the individual, a substantial amount of the literature has been focused on self-control (see Steel, 2007, for a review). An area of music research where such insights could prove to be beneficial is motivation in music education. Although learning a musical instrument can be a personally satisfying and meaningful activity, it requires considerable effort in the form of regular practice. For many music students, this may be difficult due to a lack of intrinsic motivation, belief in their competence, or reaction to the learning environment (see Renwick & Reeve, 2012, for a review). Therefore, for impatient students, the short-term temptation of not practising may be more desirable than the long-term goal. Here, we offer two proposals. First, we recommend that music education researchers incorporate theoretical frameworks used in behavioural economics to model time preferences, for example, procrastination models to measure the extent to which individuals are present-biased as well as how aware they are of their self-control problems (O’Donoghue & Rabin, 1999). This could allow music researchers to gain a better understanding of how individuals vary in their music-related motivation, and to investigate whether such model parameters can be reliable trait markers of the efficacy of music learning (see Peters & Büchel, 2011, for a review). Second, to help improve motivation in music practice, we propose the application of interventions successfully applied in other domains of self-control, such as episodic future thinking and precommitment devices (e.g., Ariely & Wertenbroch, 2002; Koffarnus et al., 2013).

### The BEM research programme

From our discussion of the extant literature and explorations of future work, we have demonstrated the benefits of applying behavioural economics to music-decision research. We have shown that by relaxing the rationality assumptions of *homo economicus* and drawing from interdisciplinary insights, researchers are able to follow an approach that is both empirically supported and wider in scope. Furthermore, by incorporating behavioural economic models, based upon principles of optimisation and underpinned by axiomatic foundations, this provides an internally consistent body of theory to work within. Here, we propose the BEM—an interdisciplinary research programme that utilises the behavioural economics toolkit for decisions related to music. Alongside these advantages, we discuss two further benefits that show the future potential of the BEM. First, while our discussion has focused on how behavioural economics can help inform areas of music-decision-making separately, the BEM could be useful in connecting seemingly disparate areas of music. For example, through the systematic review and discussion of future directions, we have identified the application of heuristics in both music listening and performance. A logical next step would be to use these commonalities to develop a more unified understanding of the psychological processes that govern both listeners and musicians. Second, as shown throughout this article, the BEM has strong practical applications in addressing real-world music issues, from tackling piracy to improving motivation in music education. We emphasise that BEM research areas are not required to be applied in isolation of each other nor are they limited to those discussed in this review. As an example, below we show how researchers can apply several BEM areas together to address a real-world music issue.

One area of concern among classical music organisations is the lack of socioeconomic diversity in the audience for classical music concerts, especially from young people and ethnic minorities (see Chan et al., 2008; DiMaggio & Mukhtar, 2004; Kolb, 2001). Barriers to attendance often cited include perceived lack of knowledge, feeling of not belonging to the community, and the desire for more social interaction at concerts (Dearn & Price, 2016; Dobson & Pitts, 2011; Kolb, 2000). Applying audience development strategies based on a combination of areas within behavioural economics may help to reduce these barriers. These include measures aimed to change social norms associated with classical concerts (e.g., more accessible music venues, relaxation of dress code, promotion of a social community through increased performer/audience interactions), framed advertising to actively challenge music stereotyping appealing to minority groups, and the use of social networks to encourage positive peer effects. Furthermore, while there has been some limited discussion about the perceived risk associated with the decision to attend concerts (Baker, 2000; Price, 2017), this area could be developed by applying theories of reference-dependent utility (Kahneman & Tversky, 1979; Kőszegi & Rabin, 2006). Here, risk attitudes of attenders can be captured relative to a reference point, such as expectations of enjoyment, and could be particularly useful to model behaviour of new-attenders who may differ in their expectations to those who attend concerts regularly.

## Conclusion

Departing from historically disconnected research programmes in psychology and economics, this article has discussed the benefits of using behavioural economics in music-decision research. Our contributions to the literature are twofold. First, through a systematic literature review, we identified 32 studies that applied behavioural economics to music-related decision-making, categorised within four research areas—heuristics and biases, social decision-making, behavioural time preferences, and dual-process theory. These studies utilised theoretical and empirical tools of behavioural economics, covering a wide area of music research, including music consumption and piracy, music preferences, music performance, music perception and memory, and music and health. Second, based on the results of our review, we discussed how behavioural economics can help develop new avenues of research. From this, we proposed *the* BEM, an interdisciplinary research programme positioned at the intersection of music, psychology, and economics. We are truly excited about such a programme, and we hope that this discussion has stimulated interest in the application of using behavioural economics to address key issues in music-decision research.

## Supplemental Material

sj-docx-1-qjp-10.1177_17470218221113761 – Supplemental material for The Behavioural Economics of Music: Systematic review and future directions Click here for additional data file. Supplemental material, sj-docx-1-qjp-10.1177_17470218221113761 for The Behavioural Economics of Music: Systematic review and future directions by Manuel Anglada-Tort, Nikhil Masters, Jochen Steffens, Adrian North and Daniel Müllensiefen in Quarterly Journal of Experimental Psychology

## Notes

1 This article solely focuses on decisions related to music. While not covered here, we acknowledge other bodies of literature that examine either the influence of music on non-music-related decisions (e.g., North et al., 2016; Palazzi et al., 2019) or where the discussion of music is secondary to broader phenomena (e.g., general consumer behaviour).

2 We excluded three keywords (uncertainty, self-control, and reference point), as these were too general for the database search and led to a large number of irrelevant search results.

3 In the model, an individual’s utility function increases not only in material payoffs as in classical game theory, but also in psychological payoffs such as the beliefs of other individuals’ kindness. See Rabin (1993) and Dufwenberg and Kirchsteiger (2004).

4 Similar to the model by Fehr and Schmidt (1999), an individual’s utility function is augmented with a fairness parameter that represents disadvantageous inequity.

5 The public goods game is an established tool in behavioural economics, used to examine contribution behaviour to a common pot within a group of players. Although the game-theoretic prediction is to contribute zero to the public good and free ride on the contributions of other players, there is a large amount of experimental evidence demonstrating that players give positive contributions. See Chaudhuri (2011) for a review.

6 While an exponential function gives a constant discount rate over time that is independent of the time period in which it is evaluated, the discount rate of a hyperbolic function does depend on the current time period and is heavily weighted for very short horizons allowing for present biased time preferences.

7 For a discussion about the role of music as a primary reinforcer, see Juslin (2019), Chapter 18.

8 Here, the assumption of rationality in the form of Nash equilibrium is relaxed such that although players play their best responses, their beliefs about other players’ actions are not required to be correct.
