# Supplementary material for: The Behavioural Economics of Music: Systematic review and future directions
Source: Q J Exp Psychol (Hove). 2022 Aug 16;76(5):1177–94. doi: 10.1177/17470218221113761 (PMC10119905; doi:10.1177/17470218221113761)
Supplement: sj-docx-1-qjp-10.1177_17470218221113761 – Supplemental material for The Behavioural Economics of Music: Systematic review and future directions [file sj-docx-1-qjp-10.1177_17470218221113761.docx]

Supplementary Material for:

**The Behavioural Economics of Music:**

**Systematic Review and Future Directions**

**Manuel Anglada-Tort, Nikhil Masters, Jochen Steffens, Adrian North, and Daniel Müllensiefen**

Behavioural economics keywords used in the systematic literature review

| Affect heuristic | Fast and frugal heuristic | Perceptual fluency |
| --- | --- | --- |
| Allais paradox | Fast-and-frugal heuristic | Preference axioms |
| Allais problem | Fisher indifference curve | Preference for fairness |
| Anchoring and adjustment | Framing effect | Preference for reciprocity |
| Anchoring heuristic | Gambler's fallacy | Present bias |
| Anchoring-and-adjustment | Game theory | Price illusion |
| Asymmetric dominance | Hedonic editing | Probability judgement |
| Attraction effect | Hedonic-editing | Probability judgment |
| Attribute substitution | Heuristic judgement | Processing fluency |
| Availability heuristic | Heuristic judgment | Projection bias |
| Axioms of preference | Heuristic of anchoring | Prospect theory |
| Biases-and-heuristics | Heuristic of availability | Psychological accounting |
| Bayesian updating | Heuristic of representativeness | Public goods game |
| Beauty contest game | Heuristics and biases | Rational choice |
| Behavioral economics | Heuristics-and-biases | Rationality assumption |
| Behavioural economics | Hyperbolic discounting | Reason based choice |
| Biases and heuristics | Infinite regress problem | Reason-based choice |
| Bounded rationality | Intertemporal choice | Reciprocity preference |
| Choice architecture | Intuitive judgement | Recognition heuristic |
| Choice bracketing | Intuitive judgment | Representativeness heuristics |
| Choice under certainty | Judgemental heuristic | Risk aversion |
| Choice under uncertainty | Judgmental heuristic | Riskless choice |
| Confirmation bias | Keynesian beauty contest | Risky choice |
| Confirmatory bias | Law of small numbers | Social preference |
| Conjunction fallacy | Level-k model | Status quo bias |
| Decoy effect | Loss aversion | Subjective expected utility |
| Delay discounting | Loss-aversion | Sunk cost |
| Dictator game | Mental accounting | System 1 and system 2 |
| Dual process | Money illusion | Temporal discounting |
| Dual-process | Monte Carlo Fallacy | Time discounting |
| Economic behavior | Nash equilibrium | Time inconsistency |
| Economic behaviour | Neoclassical economics | Time preference |
| Ellsberg paradox | Neoclassical model | Trust game |
| Ellsberg problem | Neuroeconomics | Ultimatum game |
| Endowment effect | Nudge | Utility function |
| Expected utility | Overconfidence | Value function |
| Experimental economics | Peak and end rule | Weighted utility |
| Exponential discounting | Peak end rule |  |
| Fairness preference | Peak-end rule |  |
